# Circulating *Bmi-1 *mRNA as a possible prognostic factor for advanced breast cancer patients

**DOI:** 10.1186/bcr1760

**Published:** 2007-08-21

**Authors:** Javier Silva, Vanesa García, José M García, Cristina Peña, Gemma Domínguez, Raquel Díaz, Yolanda Lorenzo, Alicia Hurtado, Antonio Sánchez, Félix Bonilla

**Affiliations:** 1 Department of Medical Oncology, Hospital Universitario Puerta de Hierro, C/San Martín de Porres, 4, E-28035 Madrid, Spain

## Abstract

**Introduction:**

Deregulation of Polycomb member Bmi-1 is involved in cell proliferation and human oncogenesis. Modulation of Bmi-1 is found in several tumor tissues, including primary breast carcinomas; however, analysis of Bmi-1 in plasma of cancer patients has not been reported. This is the first study that evaluates Bmi-1 in plasma by using a large series of primary breast carcinomas to investigate the presence at diagnosis of detectable *Bmi-1 *mRNA in plasma and possible correlations between this event and a series of clinical-pathological parameters of the tumors.

**Methods:**

*Bmi-1 *expression levels were quantified in plasma of 111 breast cancer patients and in 20 healthy controls by real-time quantitative polymerase chain reaction.

**Results:**

Cancer patients with the presence of *Bmi-1 *mRNA in plasma had higher levels of *Bmi-1 *expression than healthy controls with *Bmi-1 *mRNA in plasma. The higher expression levels of *Bmi-1 *correlated with well-established markers of poor clinical outcome in breast cancer such as positive p53 immunostaining and negative progesterone receptors. Moreover, we described for the first time a statistically significant correlation between *Bmi-1 *expression in plasma of breast cancer patients and disease-free and overall survival in advanced stages.

**Conclusion:**

Our results suggest that levels of *Bmi-1 *expression may be a surrogate marker of poor prognosis and may become clinically useful as noninvasive diagnostic markers.

## Introduction

Early detection of breast cancer can improve survival, and molecular techniques designed to detect metastases or recurrent disease in preclinical or presymptomatic phases may contribute to this strategy. Increased levels of circulating nucleic acids (CNAs) in the plasma or serum of cancer patients have been described [1]. In addition, patients with metastases have higher CNA levels than patients with localized disease [2]. Genetic and epigenetic tumor-associated DNA alterations have been found in plasma of patients with different types of cancer [3-6], which is related to poor prognosis, disease-free survival (DFS), and overall survival (OS) [7-10]. Furthermore, tumor-associated mRNA in plasma is also found in several types of cancer, such as melanoma and breast, colon, and nasopharyngeal carcinomas [11-17]. We reported previously that mRNA-based amplification methods offered higher specificity and sensitivity than DNA in plasma of breast cancer patients [18]. Additionally, an association between tumor mRNA in plasma and pathological parameters compatible with more aggressive tumors was observed [13,15,16]. Therefore, the presence of tumor RNA in plasma could lead to the development of a noninvasive prognostic method and prognostic tool that would be useful during patients' follow-up and treatment monitoring.

*Bmi-1 *is the first functional mammalian Polycomb group (PcG) proto-oncogene to be recognized. The PcG consists of several proteins that form multiprotein complexes that regulate gene activity at the chromatin level. They were initially identified as part of the memory system that ensures the faithful transmission of cell identities throughout cell division [19]. Although PcG protein expression is tightly regulated in normal cell proliferation and differentiation, it is often deregulated in several types of human cancer [20]. Several PcG genes, including *Bmi-1*, regulate the self-renewal of specific stem cell types, suggesting a link between the maintenance of cell homeostasis and carcinogenesis [21,22].

*Bmi-1 *was initially identified as an oncogene involved in the development of mouse pre-B-cell lymphomas cooperating with *c-Myc*. In mice and *in vitro *studies have indicated that Bmi-1 protein regulates the *INK4a/ARF *locus, which encodes the two tumor suppressors, p16INK4a and p19ARF (p14ARF in humans), which act in pRb and p53 cell cycle control pathways, respectively. In the absence of Bmi-1, p16INK4a may be upregulated, resulting in hypophosphorylated pRb and leading cell cycle arrest, senescence, or apoptosis, depending on the context. In contrast, deregulation of p16INK4a by Bmi-1 involves pRb hyperphosphorylation, which allows cell cycle progression. p19ARF prevents the degradation of p53 by sequestering the p53-inhibitor MDM2, thereby allowing p53-mediated cell cycle arrest and apoptosis [23-25]. Further evidence for the oncogenic role of Bmi-1 was its activation of human telomerase reverse transcriptase (hTERT), which extended the replicative life span and immortalized mammary epithelial cells, suggesting a potential regulation of telomerase activity by Bmi-1 in human breast carcinogenesis [26]. Despite the above-mentioned evidence supporting the repression of the *INK4a/ARF *failsafe mechanism or the activation of hTERT by Bmi-1, these associations have not been observed in human breast tumors [27].

Nevertheless, Bmi-1-altered expression has frequently been described in human tumors, mainly in hematological malignancies [28-33]. Disturbed Bmi-1 expression has also been reported, and correlates with poor prognosis parameters, in solid tumors such as lung cancers [34], medulloblastomas [35], neuroblastomas [36], liver [37], breast [38], colon [39], nasopharyngeal [40], and prostate [41] carcinomas.

Despite the above, no analysis of Bmi-1 in plasma of cancer patients has been reported. We evaluated *Bmi-1 *mRNA in plasma from 111 primary breast carcinomas to investigate the presence at diagnosis of detectable *Bmi-1 *mRNA in plasma and possible correlations between this event and the specific pathological and clinical parameters of tumors. Moreover, we described for the first time a statistically significant correlation between *Bmi-1 *expression in plasma of breast cancer patients and survival in advanced stages.

## Materials and methods

### Plasma samples and mRNA isolation

Informed consent was obtained from all participants after an explanation of the nature of the study, as approved by the research ethics board of our hospital. Between August 2001 and November 2003, blood samples (20 ml) were taken from 111 patients with primary breast carcinoma by venipuncture before intervention on the day of surgery: the first several milliliters were discarded to eliminate skin-plug contamination. Blood samples from 20 healthy blood donors were also obtained at the hematology unit of our hospital. Plasma was prepared by centrifugation of peripheral blood at 2,500 rpm for 25 minutes and divided into aliquots, which were snap-frozen at -80°C until processing. mRNA was extracted from 1 ml of plasma sample by Dynabeads mRNA DIRECT Kit (Dynal Biotech ASA, Oslo, Norway). Plasma was incubated with 200 μl of Dynabeads Oligo (dT) for 10 minutes at room temperature. mRNA was eluted in 10 mM Tris-HCl.

### Clinical-pathological parameters

The following parameters were obtained from the medical records of the 111 patients: age, tumor size, tumor histology, lymph node metastases, presence of steroid receptors (estrogen and progesterone), clinical stage, histological grade, proliferation index, erbB2 expression, vascular invasion, p53 immunoassay status, and presence of metastases. The clinical stage was assessed using the tumor-node-metastasis classification. The steroid receptor content was determined by an immunohistochemical procedure. The proliferation index was calculated by the Ki-67 antigen (Immunotech, Westbrook, ME, USA) in immunohistochemistry analyses. c-erbB2 expression was evaluated by a monoclonal mouse antibody (CB11; Novocastra Laboratories Ltd., Newcastle, UK). Immunohistochemistry of p53 was analyzed with the cl 1801 mouse monoclonal antibody (Oncogene Science, now part of Siemens AG, Munich, Germany) on the basis of its ability to detect up to 89% of p53 point mutations [42].

### Real-time polymerase chain reaction analysis

**Figure 1:**
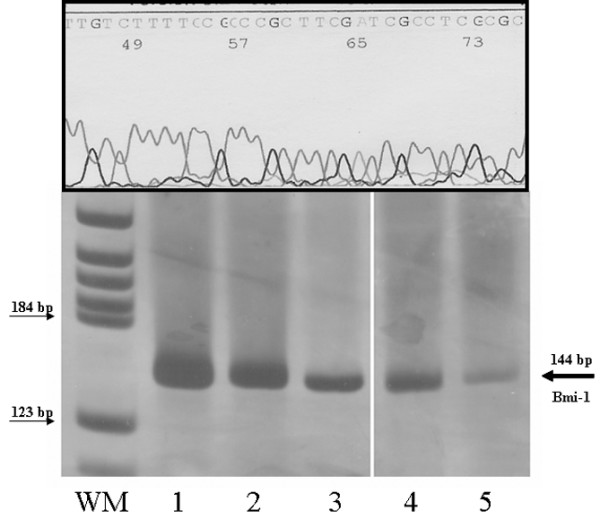
Size and sequence of specific *Bmi-1 *expression. Lanes 1 to 3 show serial dilution of the tumor cDNA used to generate standard curve (1:5, 1:25, and 1:125). Lanes 4 and 5 show *Bmi-1 *expression in the plasma of a breast cancer patient and in the plasma of a healthy control, respectively. bp, base pairs; WM, weight marker.

*Bmi-1 *expression levels were quantified in plasma of the 111 breast cancer patients and the 20 healthy controls by real-time quantitative polymerase chain reaction (PCR). *Bmi-1 *mRNA levels were standardized using ubiquitin C (*UBC*) as a reference housekeeping gene. The relative concentrations of the target and the reference gene were calculated by interpolation by means of a standard curve of each gene plotted from the same serial dilution of cDNA from tumor tissue. For the synthesis of first-strand cDNA, mRNA was reverse-transcribed using the Gold RNA PCR Core Kit (PE Biosystems, Foster City, CA, USA) in accordance with the manufacturer's instructions. Random hexamers were used as primers for cDNA synthesis. Although DNA does not bind to the beads, we designed specific intron-spanning primers for reverse transcription (RT)-PCR to avoid possible contamination. The cases in which housekeeping mRNA was not found were eliminated from the study. RT-PCR was performed in a Light-Cycler apparatus (Roche Diagnostics GmbH, Mannheim, Germany) using the LightCycler-FastStart DNA Master^PLUS ^SYBR Green I Kit (Roche Diagnostics GmbH) according to the manufacturer's instructions. Each reaction was performed in a final volume of 20 μl containing 2 μl of the cDNA product sample, 3 mM MgCl_2_, 0.5 μM of each primer, and 1× reaction mix, including FastStart DNA polymerase, reaction buffer, dNTPs, and SYBR green. The primers and conditions used are described in Table 1. Thermal cycling for both genes was initiated with a denaturation step of 95°C for 10 minutes, followed by 30 to 35 cycles (denaturation at 94°C for 2 seconds, specific annealing temperature 5 seconds, and elongation at 72°C for 5 seconds, in which fluorescence was acquired). At the end of the PCR cycles, melting curve analyses and electrophoresis of the products on nondenaturing 8% polyacrylamide gels, together with a molecular weight marker (DNA Molecular Weight Marker V; Roche Diagnostics GmbH), were run to validate the generation of the expected specific PCR product (144 base pairs). The allelic band intensity on the gels was detected by nonradioisotopic means using a commercially available silver staining method. The bands were sequenced in an ABI Prism™ 377 DNA sequencer apparatus (PE Applied Biosystems Foster City, CA, USA) (Figure 1).

**Table 1:** Primer sequences and temperature conditions for real-time quantification analysis

Primer	Sequence	Annealing temperature (°C)
BmiQ-F	GCTTCAAGATGGCCGCTTG	65
BmiQ-R	TTCTCGTTGTTCGATGCATTTC	
BmiQ-F2	CATTGTCTTTTCCGCCCGC	63
BmiQ-R2	CAAAGCACACACATCAGGTGGG	
UbcQ-F	ATTTGGGTCGCGGTTCTTG	59
UbcQ-R	TGCCTTGACATTCTCGATGGT	

### Patient follow-up

Clinical follow-up after surgery and diagnosis was based on periodic visits (every 3 months during the first year, every 6 months during the second year, and then yearly until relapse in our medical oncology department, complemented by other periodic controls in health centers of our hospital) and on clinical, biochemical, and imaging techniques (chest x-ray, bone scan, and other areas as clinically indicated). Ultrasonic study was performed when liver function was impaired. OS and DFS, defined as the period from time of diagnosis until death and the interval between diagnosis and first recurrence, respectively, were the study endpoints. Follow-up data were obtained from 98 patients.

### Data analysis

*Bmi-1 *expression data were not normally distributed (Kolmogorov-Smirnov test). Due to the non-normal distribution of the expression data, both healthy and patient samples were contrasted with *Bmi-1 *expression data in plasma by the Kruskal-Wallis test. When *Bmi-1 *expression was not detected, one of 10 equal parts of the minimum value detected in the series was assigned. Statistical analysis was performed using the SPSS package, version 11.0 (SPSS Inc., Chicago, IL, USA).

## Results

### *Bmi-1 *mRNA expression levels

**Figure 2:**
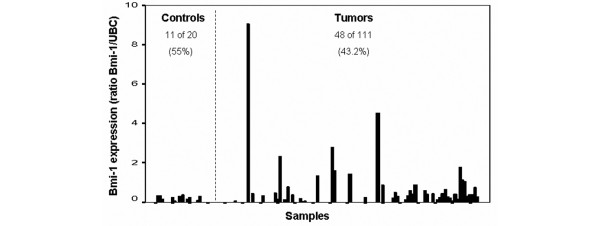
Differences between *Bmi-1 *expression levels in healthy controls and tumor samples. UBC, ubiquitin C.

mRNA of housekeeping gene *UBC *was detected by real-time PCR in the plasma of all samples. *Bmi-1 *mRNA in plasma was identified in 48 of the 111 breast cancer samples (43.2%) and in 11 of the 20 healthy controls (55%). To validate the *Bmi-1 *expression results, we analyzed the mRNA *Bmi-1 *expression in a series of 10 mRNA samples from plasma of tumor patients included in the study with a new set of optimized primers. When we analyzed the expression with the original primers in these 10 samples, three showed absence of *Bmi-1*, two showed low levels, two showed medium levels, and the other three showed high levels of mRNA *Bmi-1*. The mRNA *Bmi-1 *expression analysis of these samples with the new set of primers showed precisely the same results as those obtained with the current set. The primers and conditions used are described in Table 1. The Kruskal-Wallis test showed that cancer patients with the presence of *Bmi-1 *mRNA in plasma showed a tendency to have higher levels of *Bmi-1 *expression than healthy controls with *Bmi-1 *mRNA in plasma (*p *= 0.07) (Figure 2). Interestingly, when we compared *Bmi-1 *mRNA levels (taking into consideration only the positive cases in both populations), the Kruskal-Wallis test showed a *p *value with statistical significance (*p *= 0.019) (Table 2).

**Table 2:** Kruskal-Wallis analysis of *Bmi-1 *mRNA in plasma from positive healthy controls and breast cancer patients

Population	Number	Minimum	Maximum	Median	*P *value
Healthy controls	11	0.055	0.387	0.254	0.019
Breast cancer patients	48	0.041	9.054	0.411	

### Correlation between *Bmi-1 *mRNA expression and clinical-pathological parameters

When we correlated gene expression with clinical-pathological features of the tumors, some analyzed parameters in primary breast cancer samples showed significant associations with *Bmi-1 *mRNA expression levels in plasma. Thus, higher expression levels of *Bmi-1 *correlated with positive p53 immunostaining and with negative progesterone receptors (Table 3).

**Table 3:** Associations between *Bmi-1 *expression in plasma and clinical-pathological characteristics

Parameter	Number	Mean rank	*P *value
Age			
More than 50 years	66	57.20	NS
Less than 50 years	42	50.26	
Histology			
Invasive ductal carcinoma	79	52.05	NS
Invasive lobular carcinoma	12	54.08	
Other	10	39.00	
Vascular invasion			
Yes	16	55.69	NS
No	85	50.12	
Tumor size			
More than 2 cm	54	51.41	NS
Less than 2 cm	44	47.16	
Lymph node metastases			
Yes	45	47.80	NS
No	52	50.04	
Histological grade			
I	18	41.94	NS
II	30	37.67	
III	33	43.52	
Estrogen receptor			
Positive	74	47.15	NS
Negative	24	56.75	
Progesterone receptor			
Positive	73	46.10	0.027
Negative	25	59.44	
c-erbB2			
Positive	27	34.63	NS
Negative	37	30.95	
p53			
Positive	26	56.98	0.011
Negative	66	42.37	
Ki-67			
Positive	67	48.74	NS
Negative	27	44.43	
Metastases			
Yes	3	58.17	NS
No	95	49.23	
Stage			
I	26	54.50	
II	54	46.59	NS
III	15	49.67	
IV	3	57.67	

NS, not significant.

### Correlation between *Bmi-1 *mRNA expression and disease-free and overall survival

A total of 96 patients with survival data were followed up with for 54 months, and during this period 20 (20.4%) recurrences were observed. Kaplan-Meier analysis of our series showed that in the first year of follow-up, DFS was 93.5% (95% confidence interval [CI], 87.62% to 99.38%); in the second, DFS was 85.6% (95% CI, 77.76% to 93.44%); in the third, it was 81.2% (95% CI, 73.36% to 89.04%); and in the fourth year, it was 68% (95% CI, 50.36% to 85.64%).

**Figure 3:**
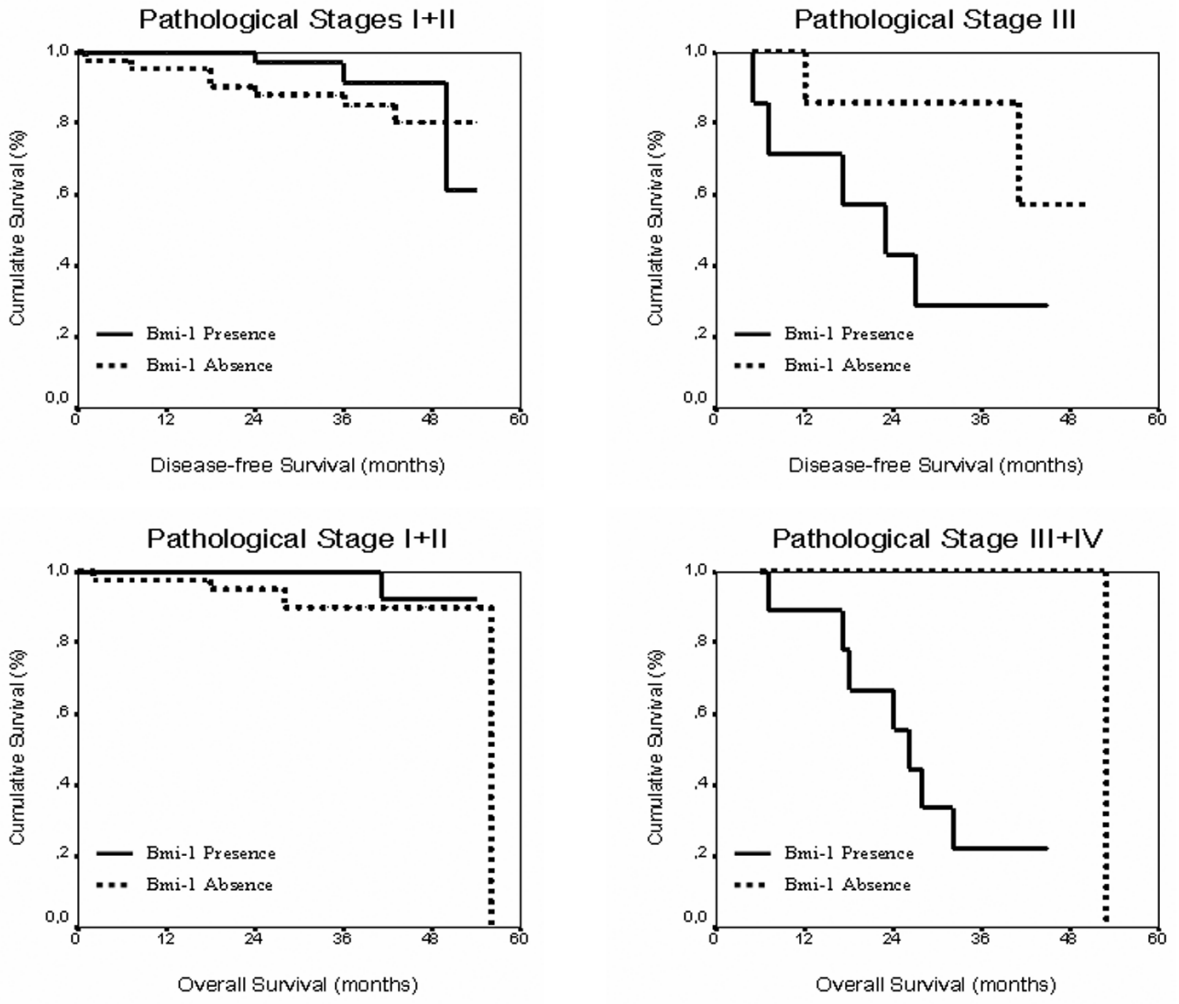
Kaplan-Meier survival curves analyzed by stages for the influence of *Bmi-1 *presence in plasma on disease-free and overall survival.

Patients were divided into two groups for the detection of *Bmi-1 *mRNA in plasma. Thus, the plasma of breast cancer patients had the absence or presence of *Bmi-1 *expression. When DFS was analyzed by Kaplan-Meier in relation to *Bmi-1 *expression, no statistically significant values were observed (*p *= 0.41). Similarly, OS was not statistically significant for expression of mRNA levels of *Bmi-1 *in plasma (*p *= 0.13). In addition, we included other clinical-pathological parameters in the analysis to make the study more comprehensive. Interestingly, when DFS was analyzed in relation to the clinical stage of the tumors concomitantly with the presence or absence of *Bmi-1*, a trend to statistically significant association between the presence of *Bmi-1 *and more advanced stages of the disease was observed (*p *= 0.08). Similarly, when we analyzed OS, including the clinical stage of the tumors, a statistically significant effect of *Bmi-1 *presence in plasma was observed (*p *= 0.0032). Differences in DFS and OS when we compared the absence/presence of *Bmi-1 *in clinical stages I+II versus stage III are described in Table 4 and Figure 3.

**Table 4:** Analysis of the association between plasma *Bmi-1 *expression levels and disease-free or overall survival in breast cancer patients regarding pathological stages

Disease-free survival	*Bmi-1 *absence	*Bmi-1 *presence	*Bmi-1 *absence	*Bmi-*1 presence
	Stages I+II	Stages I+II	Stage III	Stage III
	*N *= 43	*N *= 36	*N *= 8	*N *= 7
Cumulative survival				
First year	95.2% (89.3%–100%)	100%	85.7% (60.2%–100%)	71.4% (45.9%–96.8%)
Second year	87.9% (78.1%–97.7%)	96.8% (90.9%–100%)	85.7% (60.2%–100%)	42.8% (5.5%–80.1%)
Third year	84.9% (73.1%–96.6%)	91.4% (79.6%–100%)	85.7% (60.2%–100%)	28.5% (0%–61.8%)
Fourth year	80.2% (66.5%–93.9%)	91.4% (79.6%–100%)	57.1% (11.1%–100%)	28.5% (0%–61.8%)
56 months	80.2% (66.5%–93.9%)	61.1% (12.1%–100%)	57.1% (11.1%–100%)	28.5% (0%–61.8%)

Significance	NS		0.08	

Overall survival	*Bmi-1 *absence	*Bmi-1 *presence	*Bmi-1 *absence	*Bmi-1 *presence
	Stages I+II	Stages I+II	Stages III+IV	Stages III+IV
	*N *= 43	*N *= 36	*N *= 8	*N *= 9

Cumulative survival				
First year	97.5% (93.6%–100%)	100%	100%	88.8% (69.2%–100%)
Second year	95.1% (89.2%–100%)	100%	100%	55.5% (25.2%–86.8%)
Third year	89.9% (80.1%–99.7%)	100%	100%	22.2% (0%–49.6%)
Fourth year	89.9% (80.1%–99.7%)	92.3% (78.6%–100%)	100%	22.2% (0%–49.6%)
56 months	0%	92.3% (78.6%–100%)	0%	22.2% (0%–49.6%)

Significance	NS		0.0032	

Values in parentheses are 95% confidence intervals. NS, not significant.

## Discussion

The PcG protein Bmi-1 is a transcriptional repressor that has been involved in axial patterning, hematopoiesis, regulation of cell proliferation, senescence, and regulation of stem cell self-renewal. Bmi-1 expression is often deregulated in several types of human cancer, including hematological and solid tumors, which correlate with poor prognosis parameters. Moreover, Bmi-1 has recently been shown to be a useful molecular marker for predicting occurrence and prognosis in high-susceptibility cancer syndromes such as myelodysplastic syndrome [43]. The main line of evidence involving Bmi-1 in tumorigenesis is the repression of INK4a/ARF suppressor proteins, deregulating both pRb and p53 cell cycle control pathways, facilitating cell proliferation, and desensitizing cells to apoptosis. However, the effect of Bmi-1 overexpression on the inactivation of the *INK4a/ARF *transcripts in human breast cancer is unclear, as we reported previously [27].

Although all studies analyzing the deregulated expression of Bmi-1 have been conducted in primary tumors, no data on Bmi-1 in plasma of cancer patients have been reported. Thus, we attempted to detect *Bmi-1 *mRNA in plasma from a large series of 111 patients with primary breast carcinomas to assess its possible value as a prognostic marker and its applicability as a noninvasive tool for prognosis.

*Bmi-1 *mRNA in plasma detected by real-time PCR was identified in 43.2% of the breast cancer samples and in 55% of the healthy controls. The presence of free CNAs has been observed in healthy controls [1,2], and *Bmi-1 *expression has been reported in nontumor tissue thought to be at lower levels than tumor tissues [37,38]. Thus, it was not surprising that we found *Bmi-1 *mRNA in plasma of healthy controls. Nevertheless, although the frequency of detection was slightly higher in the healthy population than in cancer patients, the values of mean rank and statistical significance obtained in the Kruskal-Wallis test showed that *Bmi-1 *mRNA expression levels were higher in plasma from breast carcinoma patients than *Bmi-1 *mRNA expression levels in plasma from healthy controls. Furthermore, high expression levels of *Bmi-1 *correlated with several clinical-pathological features of the tumors. Positive nuclear immunostaining of p53 (suggestive of p53 mutation) and negative progesterone receptor immunohistochemistry are well-established markers of poor clinical outcome in breast cancer. Both alteration markers were statistically associated with the higher expression levels of *Bmi-1 *in plasma. We previously reported a correlation between expression of *Bmi-1 *and p53 and steroid receptor immunostaining in primary breast tumors [27]. Interestingly, in this previous study, we found that *Bmi-1 *overexpression was associated with negative p53 and positive progesterone receptor staining, which clearly showed differences between cancer cells in primary tumor and CNA-free cells in plasma. Our results showed that increased *Bmi-1 *expression measured in CNA-free cells in plasma was associated with more malignant conditions in the primary location of tumor, such as altered p53 cell cycle-regulating pathway and a more undifferentiated stage.

Initially, our data show no correlation between the presence of *Bmi-1 *mRNA detected in plasma of breast cancer patients and shorter DFS or OS evaluated by Kaplan-Meier analysis. Nevertheless, we observed a statistically significant association between the presence of *Bmi-1 *in plasma and survival when we included the clinical stage in the analysis. In fact, this stratified analysis showed a trend to statistical significance between lower DFS in those patients with the presence of *Bmi-1 *in plasma in more advanced stages of the disease and the remainder. More statistically significant effects of the presence of *Bmi-1 *in plasma in advanced stages were observed when OS was analyzed. The finding that the presence of *Bmi-1 *in plasma in advanced clinical stages predicted OS with more statistical efficiency than DFS suggests that *Bmi-1 *may be involved in the aggressiveness of the disease rather than in the time of relapse.

In conclusion, in the present study, we examined the mRNA levels of *Bmi-1 *in plasma in a large series of primary breast carcinomas. To our knowledge, this is the first study on the expression of oncogene *Bmi-1 *in plasma of cancer patients. We found associations between high levels of *Bmi-1 *and several clinical-pathological parameters for poor prognosis, such as positive p53 and negative progesterone receptor staining. Moreover, we associated the mRNA presence of this PcG gene in plasma with worse survival at a more advanced clinical stage, indicating that this may affect the outcome of the disease. Our results from the correlative analysis suggested that the presence of *Bmi-1 *mRNA in plasma of patients with breast tumors may be a possible prognostic marker, which can be obtained by a noninvasive method.

## Conclusion

Early detection of breast cancer is critical for improving survival of patients. Techniques designed to detect metastases or recurrent disease in preclinical states may contribute to this purpose. We evaluated for the first time the oncogene Bmi-1 in plasma in a large series of primary breast carcinomas to investigate the presence at diagnosis of detectable *Bmi-1 *mRNA in plasma and its possible correlations with clinical-pathological parameters of the tumors and survival of patients. Our results suggest that levels of *Bmi-1 *expression may be a surrogate marker of poor prognosis and may become clinically useful as noninvasive diagnostic markers.

## Abbreviations

CI = confidence interval; CNA = circulating nucleic acid; DFS = disease-free survival; hTERT = human telomerase reverse transcriptase; OS = overall survival; PcG = Polycomb group; PCR = polymerase chain reaction; RT-PCR = reverse transcription-polymerase chain reaction; *UBC *= ubiquitin C.

## Competing interests

The authors declare that they have no competing interests.

## Authors' contributions

JS and VG contributed to study conception and design, collection and assembly of data, data analysis and interpretation, and manuscript writing and gave final approval of the manuscript. AH and AS contributed to provision of study materials or patients and gave final approval of the manuscript. FB contributed to provision of study materials or patients and manuscript writing and gave final approval of the manuscript. GD, RD, and YL contributed to collection and assembly of data and gave final approval of the manuscript. JMG and CP contributed to data analysis and interpretation and gave final approval of the manuscript. JS and VG are co-authors of this article.
